# High‐Yield Production of Biohybrid Microalgae for On‐Demand Cargo Delivery

**DOI:** 10.1002/advs.202001256

**Published:** 2020-07-02

**Authors:** Mukrime Birgul Akolpoglu, Nihal Olcay Dogan, Ugur Bozuyuk, Hakan Ceylan, Seda Kizilel, Metin Sitti

**Affiliations:** ^1^ Physical Intelligence Department Max Planck Institute for Intelligent Systems Stuttgart 70569 Germany; ^2^ Chemical and Biological Engineering Department Koç University Istanbul 34450 Turkey; ^3^ School of Medicine and School of Engineering Koç University Istanbul 34450 Turkey

**Keywords:** microrobots, biohybrids, microswimmers, chitosan, *Chlamydomonas reinhardtii*, microalgae, drug delivery, light‐triggered drug release

## Abstract

Biohybrid microswimmers exploit the swimming and navigation of a motile microorganism to target and deliver cargo molecules in a wide range of biomedical applications. Medical biohybrid microswimmers suffer from low manufacturing yields, which would significantly limit their potential applications. In the present study, a biohybrid design strategy is reported, where a thin and soft uniform coating layer is noncovalently assembled around a motile microorganism. *Chlamydomonas reinhardtii* (a single‐cell green alga) is used in the design as a biological model microorganism along with polymer–nanoparticle matrix as the synthetic component, reaching a manufacturing efficiency of ≈90%. Natural biopolymer chitosan is used as a binder to efficiently coat the cell wall of the microalgae with nanoparticles. The soft surface coating does not impair the viability and phototactic ability of the microalgae, and allows further engineering to accommodate biomedical cargo molecules. Furthermore, by conjugating the nanoparticles embedded in the thin coating with chemotherapeutic doxorubicin by a photocleavable linker, on‐demand delivery of drugs to tumor cells is reported as a proof‐of‐concept biomedical demonstration. The high‐throughput strategy can pave the way for the next‐generation generation microrobotic swarms for future medical active cargo delivery tasks.

## Graphical abstract

Algal microrobots, using green microalgae *Chlamydomonas reinhardtii*, are constructed with polymer–nanoparticle matrix coating. This design strategy has a high manufacturing yield of around 90%. Furthermore, a model chemotherapeutic drug (doxorubicin) is conjugated to the nanoparticles embedded in the thin coating and on‐demand light‐triggered drug release to cancer cells is demonstrated as a proof‐of‐concept.

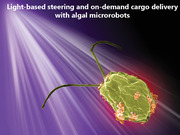

Over the past decade, biohybrid microrobots, in which living mobile microorganisms are physically integrated with untethered artificial structures, have gained growing interest to enable the active locomotion and cargo delivery to a target destination.^[^ ^1^, ^2^, ^3^, ^4^ ^]^ In addition to the motility, the intrinsic capabilities of sensing and eliciting an appropriate response to artificial and environmental changes make cell‐based biohybrid microrobots appealing for transportation of cargo to the inaccessible cavities of the human body for local active delivery of diagnostic and therapeutic agents.^[^ ^5^, ^6^, ^7^ ^]^ Active locomotion, targeting and steering of concentrated therapeutic and diagnostic agents embedded in mobile microrobots to the site of action can overcome the existing challenges of conventional therapies.^[^ ^8^, ^9^, ^10^ ^]^ To this end, bacteria have been commonly used with attached beads and ghost cell bodies.^[^ ^11^, ^12^, ^13^, ^14^, ^15^, ^16^, ^17^, ^18^ ^]^ The downside of such microorganisms includes the risks about the possibility to create pathogenicity, unavoidable rapid growth rate of bacteria in physiological conditions, and their potential antibiotic resistance. Hence, the need for using more biocompatible and slowly growing motile microorganisms compared to bacteria has increased for the development of biohybrid systems.^[^ ^2^ ^]^

*Chlamydomonas reinhardtii* (*C. reinhardtii*) is a unicellular green microalga. The wild‐type *C. reinhardtii* has a spherical shape that averages about 10 µm in diameter.^[^ ^19^ ^]^ This microorganism can perceive the visible light and be steered by it (i.e., phototaxis) with high swimming speeds in the range of 100–200 µm s^−1^.^[^ ^7^ ^]^ It has natural autofluorescence that permits label‐free fluorescent imaging.^[^ ^19^ ^]^ We have recently explored *C. reinhardtii* as the live component of biohybrid microrobots for the active delivery of therapeutics.^[^ ^7^ ^]^ They are biocompatible with healthy mammalian cells, leave no known toxins, mobile in the physiologically relevant media, and allow for surface modification to carry cargo on the cell wall.^[^ ^7^, ^20^, ^21^, ^22^, ^23^ ^]^ Alternative attachment strategies for *C. reinhardtii* have been proposed for the assembly through modifying the interacting surfaces by electrostatic interactions^[^ ^7^, ^20^ ^]^ and covalent bonding.^[^ ^24^ ^]^ Despite such elegant strategies, their manufacturing yield and efficiency are lower than expected, with only a limited population of algal cells carrying their payload. This drawback mainly stems from the low self‐assembly yield of the micrometer‐size live cells with the micrometer‐size particles, which relies on their effective random motion‐based collision in a 3D volume within an experimentally feasible time scale.^[^ ^25^ ^]^

Here, we report a biohybrid algal microswimmer system that has high manufacturing yield by molecular assembly of the nonliving component around the cell wall. The nonliving component consists of a conformal layer around *C. reinhardtii* using a natural biopolymer chitosan through electrostatic interactions, where positively charged chitosan polymer attaches to the negatively charged *C. reinhardtii* cell wall. Chitosan acts as a binding agent, which significantly increases the further attachment of nanoparticles. The thin chitosan–nanoparticle coating has no adverse effect on the motility and phototactic properties of biohybrid microalgae. As a proof‐of‐concept demonstration, biohybrid microalgae were used to perform on‐demand delivery of chemotherapeutic cargo to SK‐BR‐3 cancer cells by modifying the attached nanoparticles with doxorubicin (DOX) by means of a photocleavable linker. Overall, this work presents a high‐throughput method of manufacturing biohybrid microswimmers which forms the basis for the development of next‐generation microalgae‐based cargo delivery platform.

Strategies for coating of living cells electrostatically have taken advantage of the negative charge of the cell wall or membrane to deposit positively charged materials.^[^ ^26^ ^]^ Various microorganisms, including yeasts,^[^ ^27^ ^]^ bacterial spores,^[^ ^28^ ^]^ bacteria,^[^ ^29^ ^]^ algae,^[^ ^30^ ^]^ and pancreatic islets^[^ ^31^ ^]^ were coated with cationic polymers or particles via electrostatic interactions for diverse applications such as immunoisolation,^[^ ^32^ ^]^ magnetic modification,^[^ ^11^ ^]^ or harvesting.^[^ ^30^, ^33^ ^]^ *C. reinhardtii* has a cell wall that mainly contains pectin and glycoproteins with anionic carboxylate groups, which creates a negatively charged surface on its cell wall.^[^ ^34^ ^]^ For the preparation of biohybrid microrobots, we made use of the basic phenomenon of electrostatic interactions by introducing oppositely charged surfaces with respect to each other (**Figure** **1**A). We prepared a coating mixture consisting of: i) chitosan‐coated iron oxide nanoparticles (CSIONPs), and ii) chitosan polyelectrolyte solution. CSIONPs were dispersed in chitosan polyelectrolyte solution to prepare the coating mixture. In the dispersion, positively charged chitosan binds to the cell wall of the microalgae and assembles into a coating network. Electrostatic interactions of several polymers and nanoparticles with algal cells were previously shown for different applications.^[^ ^30^, ^33^, ^35^ ^]^ Scanning electron microscope (SEM) images of a single bare and biohybrid microalgae show the cell morphologies before and after coating (Figure 1B).

**Figure 1:**
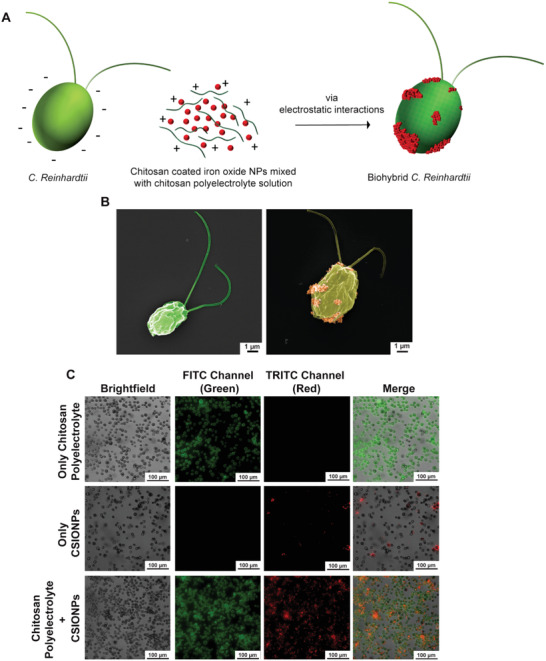
Production of biohybrid *C. reinhardtii* microswimmers. A) Schematics of production steps for biohybrid *C. reinhardtii*. B) SEM images of bare microalgae (left) and biohybrid microalgae (right) coated with chitosan‐coated iron oxide nanoparticles (CSIONPs). Images were pseudocolored. A darker green color on the right SEM image represents chitosan coating on microalgae cell wall. Orange‐colored particles represents CSIONPs. C) Microscopy images of biohybrid microalgae treated with three different solutions: 1) microalgae coated with 5 µg mL^−1^ green‐fluorescent chitosan polyelectrolyte solution (first row), 2) microalgae coated with 10 µg mL^−1^ red fluorescent CSIONPs (second row), and 3) microalgae coated with both CSIONPs (10 µg mL^−1^) dispersed in chitosan polyelectrolyte solution (5 µg mL^−1^) (third row).

Microalgae at OD_680_: 0.25–0.5 (Figure S1, Supporting Information) were incubated separately with three different solutions to evaluate the binding capability of chitosan polyelectrolyte solution. First, microalgae were treated only with green fluorescent chitosan solution (i.e., no CSIONPs), where conformal coatings around algal cells are indicated by green fluorescence (Figure 1C, first row). Next, microalgae were incubated only with red fluorescent CSIONPs (i.e., without dispersing CSIONPs in chitosan polyelectrolyte solution). In this case, a limited number of particles were attached to microalgae cell wall as demonstrated by the scarcity of red fluorescence (Figure 1C, second row). Finally, when microalgae were incubated within the mixture of CSIONPs dispersed in chitosan solution, coating was dramatically enhanced as illustrated in Figure 1C (third row), in which the population of red fluorescent algal cells is significantly elevated. Chitosan is highly cationic due to amino groups in its backbone, hence the solution can occupy all sections of the negatively charged algal cell wall and eventually resulting in an improved surface coverage. Since CSIONPs are dispersed within the cationic chitosan solution, their attachment on the cell wall of microalgae is therefore enhanced. Accordingly, all further coatings were carried out by dispersing CSIONPs in chitosan solution and incubating microalgae within the solution for 5 min.

Fluorescence imaging of a single bare and biohybrid microalgae demonstrates the chitosan coating (indicated by green fluorescent) and CSIONPs (indicated by red fluorescence) on algal cell wall (**Figure** **2**A). Coating efficiency of the algal population was determined by comparing the red fluorescent population to the total algal population. Incubation of microalgae within the coating mixture consisting of 0.01 mg mL^−1^ CSIONPs resulted in biohybrid microalgae population with a coating yield of ≈90% (Figure 2B), which is much higher than previously reported yield values.^[^ ^7^, ^20^, ^24^ ^]^ Stability of coating against time was investigated by capturing images of biohybrid microalgae at several time intervals (Figure S2, Supporting Information). We observed that even after 24 h, both green and red fluorescence colors were visible, which was an indication for the presence of both chitosan layer and CSIONPs on cell walls. The reason for reduced fluorescence with time after coating up to 24 h could be due to the doubling time of *C. reinhardtii*, which is around 12 h.^[^ ^36^ ^]^

**Figure 2:**
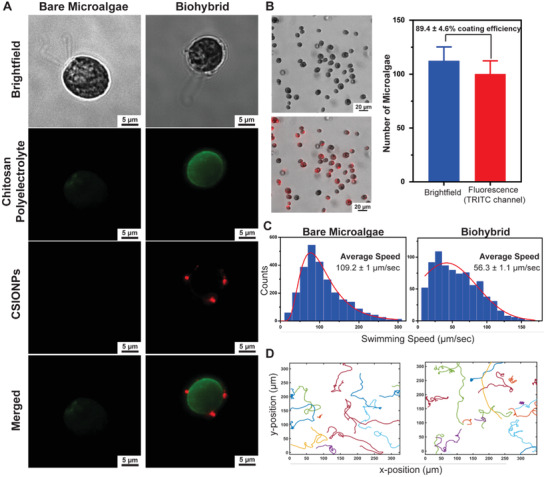
Characterization of biohybrid *C. reinhardtii*. A) Microscope images of a bare and biohybrid microalgae showing the chitosan and CSIONPs coating via green and red fluorescence, respectively. B) Coating efficiency of microalgae population. 89.4 ± 4.6% of the microalgae population expressed TRITC signal, indicating the attachment of CSIONPs on the cell wall. C) 2D swimming speed analyses of bare and biohybrid microalgae. D) 2D swimming trajectories of bare and biohybrid microalgae.

2D swimming mean velocities of bare and biohybrid microalgae are shown in Figure 2C. Bare microalgae had an average swimming speed of 109.2 ± 1 µm s^−1^, whereas average swimming speed of biohybrid microalgae was recorded as 56.3 ± 1 µm s^−1^ (Movies S1 and S2, Supporting Information). Swimming of biohybrid microalgae showing chitosan coating and CSIONPs coatings are also presented (Movies S3 and S4, Supporting Information, respectively). The decrease in swimming velocity can be attributed to the location of CSIONPs on algal cells (Figure S3, Supporting Information). It was previously shown that when micrometer‐sized beads were adhered on or close to the flagella, motility of *C. reinhardtii* cells were hindered.^[^ ^20^ ^]^ Centrifugation of algal cells may also have an adverse effect on the motility, as an average swimming velocity of ≈80 µm s^−1^ was observed when bare algal cells were centrifuged twice at 300 × *g* for 1.5 min (Figure S4 and Movie S5, Supporting Information). Although swimming velocity is decreased, there was no impediment in translation ability of biohybrid microalgae, as illustrated by swimming trajectories (Figure 2D).

Propulsion of motile microorganisms, such as bacteria, microalgae, sperm cells, and macrophages, relies on their autonomous motion governed by the motility mechanisms including crawling, contractile behavior, and flagellar or ciliary movement.^[^ ^2^, ^37^ ^]^ In the absence of an external factor, the movement of a particular microorganism is customarily random; hence, it is of vital importance for biohybrid microrobots that their motion is controllable and steerable. Controlling the motion of a biohybrid microrobot could be realized by two methods: i) intrinsically by microorganism's intelligence to exploit the surrounding chemical energy for thrusting (e.g., chemotaxis,^[^ ^38^, ^39^, ^40^, ^41^, ^42^ ^]^ pH‐taxis,^[^ ^43^ ^]^ phototaxis)^[^ ^20^, ^44^, ^45^, ^46^ ^]^, and ii) extrinsically by manipulating the artificial compartment attached to the living microorganism (e.g., magnetic^[^ ^7^, ^11^, ^47^ ^]^ or acoustic waves‐based control^[^ ^48^ ^]^).

*C. reinhardtii* is a biflagellated, phototactic green microalgae, which swim steadily towards a light stimulus due to their optical receptors.^[^ ^49^ ^]^ Light response of *C. reinhardtii* is fast; a linear phototaxis response is observed within 1–3 min after light stimulus.^[^ ^50^ ^]^ We harnessed *C. reinhardtii*’s phototactic behavior in our biohybrid platform due to its robustness, fast‐response time and innocuous nature. We investigated whether our coating strategy, in which we coat algal body with uniform layer of chitosan with scattered nanoparticles, impairs with microalgae phototaxis abilities. Light‐based steering (phototaxis) of algal cells is illustrated **Figure** **3**A. Phototaxis behaviors of bare and biohybrid microalgae were demonstrated in a microfluidic channel with axial length of 2 cm. Microfluidic channels filled with either bare or biohybrid microalgae were installed on an inverted light microscope. Without any light stimulus (at *t* = 0), the movements of algal cells are randomized by their synchronous and asynchronous beating sequences.^[^ ^51^ ^]^ When the right side of the microchannel was illuminated for 10 min, the migration and accumulation of both bare and biohybrid algal cells were observed toward the light stimulus as presented in the phase contrast images of the microchannel (*t* = 10 min) (Figure 3B, right). Next, when the light stimulus was switched to the left, both bare and biohybrid algal cells started to swim towards the new stimulus, eventually concentrating the left side of the channel (*t* = 20 min) (Figure 3B, left). Quantification of algal cells at each end of the microchannels were performed to establish a packaging density. The number of algal cells at the right and left side of microchannels were quantified and compared at 10 and 20 min time points (Figure 3C). Statistically significant number of microalgae were accumulated at the side of the light‐stimulus for both bare and biohybrid algal cells. This data presents a robust mechanism to steer biohybrid microalgae towards a light‐stimulus in a fast and controlled manner. In addition, inclusion of chitosan and nanoparticles on algal body did not reduce phototaxis response of biohybrid microalgae.

**Figure 3:**
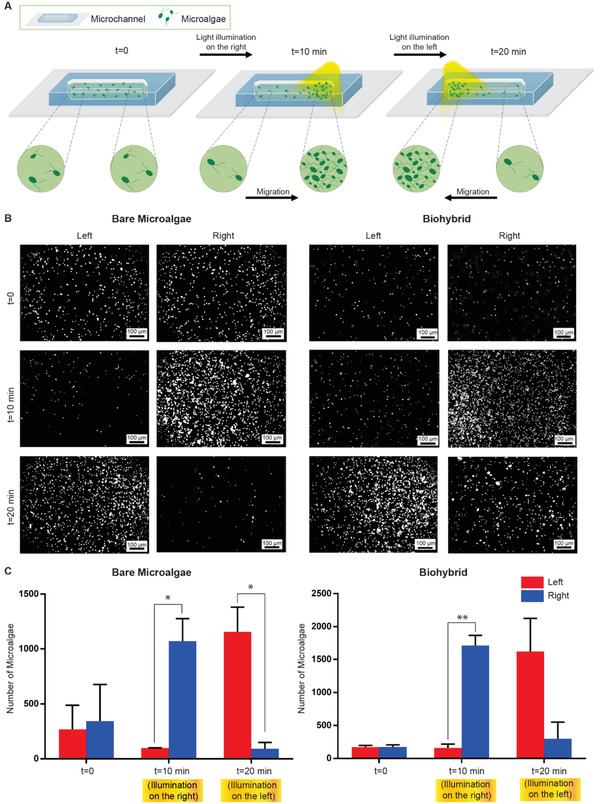
Visible light response (phototaxis) of biohybrid *C. reinhardtii*. A) Schematics of light‐driven steering (phototaxis) of microalgae under visible light. The right side of the channel was illuminated for 10 min to steer microalgae to the right. Next, the left side of the microchannel was illuminated for 10 min to steer microalgae to the left. B) Phase contrast images of bare and biohybrid microalgae after light‐driven phototactic steering (white spots correspond to the microalgae). Images were taken after 10 min (right) and 20 min (left). C) Quantification of microalgae number at each end of the microchannel. Scale bars: 100 µm.

As a proof‐of‐concept cargo delivery demonstration, we modified the nanoparticles with a model cancer drug, DOX, via a photocleavable linker and prepared biohybrid microalgae using these particles. Light‐triggered delivery systems offer on‐demand release of molecules with high spatiotemporal resolution. In such systems, therapeutics can be concentrated on a desired site upon light exposure, preventing nonspecific distribution of molecules.^[^ ^52^, ^53^, ^54^ ^]^ Through the use of on‐demand release mechanisms, therapeutic molecules can be preserved on biohybrid microswimmers, preventing possible side effects on biohybrids that might arise from unwanted release of molecules.

Here, we modified the amino groups of the CSIONPs with the model antitumor drug DOX by means of a photocleavable linker (*o*‐nitrobenzyl).^[^ ^55^ ^]^ The reaction scheme is presented in **Figure** **4**A. We used a two‐step route for the conjugation of DOX to CSIONPs: 1) NHS‐amine reaction, where amino groups of the CSIONPs were reacted with NHS groups of the *o*‐nitrobenzyl linker through NHS‐amino coupling. The resulting nanoparticles had alkyne ends tethering from the photocleavable linker chain. 2) Nanoparticles with alkyne ends were modified with DOX‐azide (DOX‐N_3_) through copper (I) catalyzed‐cycloaddition (CuAAC). As a negative control group, nanoparticles were only treated with DOX‐N_3_ as in the second step, without reacting them with linker in the first place. Since there are no alkyne ends in this group for CuAAC reaction, DOX molecules could only be physically adsorbed by nanoparticles. CSIONPs reacted with both linker and DOX are referred as CSIONPs‐L‐DOX, whereas nanoparticles reacted only with DOX are referred as CSIONPs‐DOX. After reaction steps, nanoparticles were washed several times with ddH_2_O. After each washing step, particles were centrifuged and supernatants were collected for determination of DOX concentration.

**Figure 4:**
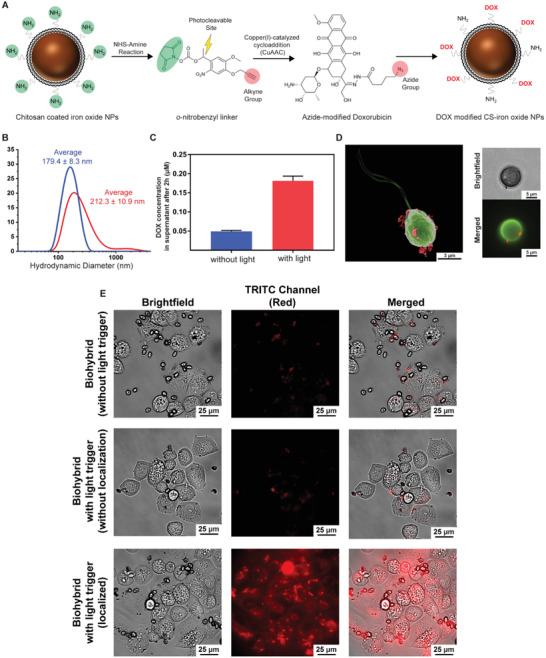
Proof‐of‐concept in vitro demonstration of drug delivery to cancer cells by biohybrid microalgae. A) Reaction scheme. *o*‐nitrobenzyl (linker, L) is conjugated to CSIONPs (nonfluorescent) through NHS‐amine coupling chemistry. Next, alkyne end of the linker is reacted with DOX‐N_3_ through copper (I)‐catalyzed cycloaddition (CuAAC) reaction. B) Hydrodynamic diameters of CSIONPs (blue) and CSIONPs‐L‐DOX (red). C) Drug release from CSIONPs‐L‐DOX with light (red), and without light (blue) after 2 h from 1 mg mL^−1^ nanoparticle solution. D) SEM, brightfield and fluorescent images of CSIONP‐L‐DOX modified biohybrid microalgae. SEM image was pseudocolored. E) Light‐triggered DOX release from the biohybrid microalgae on SK‐BR‐3 cancer cells. CSIONP‐L‐DOX modified biohybrid microalgae were incubated with SK‐BR‐3 cells for 2 h after light‐triggered drug release. After 2 h, fluorescence imaging of the cell culture was done to observe drug internalization. TRITC (red fluorescence) signal detected on SK‐BR‐3 cells indicates drug internalization by cells. No internalization was observed when light illumination was not applied. Likewise DOX uptake was not detected when the number of CSIONP‐L‐DOX modified biohybrid microalgae was very few around SK‐BR‐3 cells, although illumination was applied for 5 s. Significant drug internalization by SK‐BR‐3 cells surrounded by a larger population of CSIONP‐L‐DOX modified biohybrid microalgae was detected.

Fluorescence intensity measurements have shown that CSIONPs‐L‐DOX group had much less DOX in its supernatant, indicating that the DOX was chemically conjugated to CSIONPs (Figure S5A, Supporting Information). In addition, dispersion of CSIONPs‐L‐DOX had significantly higher fluorescence value than CSIONPs‐DOX after washing steps (Figure S5B, Supporting Information). This also confirms the conjugation of DOX molecules to the CSIONPs. Hydrodynamic diameters of the particles were characterized by dynamic light scattering (DLS). According to the DLS analysis, bare CSIONPs, and CSIONPs‐L‐DOX have 179.4 ± 8.3, and 212.3 ± 8.3 nm hydrodynamic diameters, respectively (Figure 4B). CSIONPs‐L‐DOX has slightly higher hydrodynamic diameter probably due to the conjugation of the DOX molecules on the surface. CSIONPs‐L‐DOX showed significantly higher DOX release 2 h after being exposed to light compared to the case of no light exposure (Figure 4C).

Drug loaded nanoparticles (CSIONPs‐L‐DOX) were attached on the surface of algal cell wall through electrostatic interactions as presented in Figure 1A previously. SEM, light, and fluorescence microscopy images are presented in Figure 4D. A single biohybrid microalgae in the SEM image shows dispersed attachment of CSIONPs‐L‐DOX on the algal cell wall. DOX molecules are chemically bound to CSIONPs via amino groups, which gives chitosan its cationic properties. Partial consumption of some of these amino groups with DOX did not generate loss of coating efficiency. In addition, swimming velocity of CSIONPs‐L‐DOX‐coated algal cells were measured as 67.1 ± 0.9 µm s^−1^, which is in agreement with previous velocity measurements (around 109 µm s^−1^ for bare and 56 µm s^−1^ for biohybrid microalgae) (Figure S6, Supporting Information).

For cell culture experiments, we first determined the noncytotoxic dose of microalgae to SK‐BR‐3 cancer cells. We used microalgae at OD_680_:0.6 for the upcoming experiments since it presented the highest viability for cells (Figure S7, Supporting Information). Next, for the demonstration of light‐triggered drug release, SK‐BR‐3 breast cancer cells were incubated with CSIONPs‐L‐DOX modified biohybrid microalgae. No drug internalization was observed when the biohybrid microalgae were not illuminated with light (Figure 4E, first row). In case of light‐triggered drug release group, internalization of DOX again was not clear where the number of biohybrid microalgae is few around cell clusters (Figure 4E, second row). Drug internalization was clearly observed by SK‐BR‐3 cells, where the biohybrid microalgae were localized on the cancer cells (Figure 4E, third row). We also detected the attachment of biohybrid microalgae on the cancer cells, probably due to the electrostatic interactions between positively charged coating and negatively charged membrane of SK‐BR‐3 cells.

This phenomenon also boosted the diffusion of the released drug from extracellular environment to cytoplasm more effectively, since the drug carriers and cell membranes are in direct contact (Movie S6, Supporting Information). Internalization of drug molecules in the selected target areas enables an accommodating biohybrid microrobotic platform, where microswimmers can be guided to a designated region and carry out their therapeutic task with high accuracy. Internalization of drug molecules by cancer cells prove that biohybrid microalgae can be manipulated with external factors to deliver their medical cargo with high precision. However, the on‐demand release mechanism presented in our study requires application of UV light (365 nm), which has limited penetration depth and cell viability issues.^[^ ^53^ ^]^ To this end, the optical upconversion processes, where the near‐infrared (NIR) light is converted to UV light, may be a potential solution for the realistic medical applications of UV‐triggered systems.^[^ ^53^ ^]^ Also, other stimuli‐responsive release mechanisms, such as thermal^[^ ^56^ ^]^ and ultrasound^[^ ^57^ ^]^‐based triggering methods, may be integrated to our platform described here as a future work.

In this work, we demonstrated a new noncovalently formed biohybrid design that encase microalgae with a thin and soft uniform coating. This design strategy reaches a high manufacturing yield of around 90%, far higher than the previously reported microalgal biohybrid designs. The biohybrid microalgae did not lose motility after the coating process and kept their phototactic behavior. As a proof‐of‐concept cargo delivery demonstration, we modified the coating with a model drug DOX through a photocleavable linker, and showed uptake of DOX molecules by the cancer cells upon a light‐stimuli. The high‐throughput strategy presented in this study can be applied to a broad range of microorganisms, and advance the performance of the biohybrid microrobots for different applications.

## Experimental Section

##### Materials

*C. reinhardtii* (*C. reinhardtii*) was received from Chlamydomonas Resource Center (St. Paul, MN). Chitosan fluorescein (200–500 cP, 85% DDA, excitation (Ex)/emission (Em) wavelength 494/518 nm) was obtained from Creative PEGworks (Chapel Hill, NC). Chitosan coated iron oxide NPs (Product names: nano‐screenMAG‐Chitosan, and the fluorescent particles, nano‐screenMAG‐Chitosan/red 200 nm, Ex/Em wavelength 578/613 nm) were purchased from Chemicell GmbH (Berlin, Germany). TAP medium and McCoy's 5A (Modified) Medium were obtained from Thermo Fischer Scientific. Pluoronic F‐108 and hexamethyldisilazane was purchased from Sigma‐Aldrich (St. Louis, MO). Azide‐modified doxorubicin and photocleavable *o*‐nitrobenzyl linker ((1‐(5‐methoxy‐2‐nitro‐4‐prop‐2‐ynyloxyphenyl)ethyl *N*‐succinimidyl carbonate) were obtained from LifeTein LLC (Hillsborough, NJ).

##### Culture of *C. Reinhardtii*

*C. reinhardtii* (CC‐125 wild type mt+) were grown in TAP medium at room temperature using a 12/12 h light–dark cycle (Philips MASTER TL‐D 58W/840 Super 80 Weiss) in an orbital shaker at 150 rpm. Growth curve of *C. reinhardtii* was prepared by measuring OD680 of 200 µL microalgae dispersion on several time points using a plate reader (BioTek's Synergy H1, Winooski, VT, USA).

##### Preparation of the Biohybrid Algal Microswimmers

Biohybrid algal microswimmers were prepared by incubation of the microalgae in a chitosan polyelectrolyte (with green‐fluorescent tag) solution with chitosan‐coated iron oxide NPs (CS‐iron oxide NPs, with red fluorescence tag). All coating experiments were done at growth phase of *C. reinhardtii* (OD_680_: 0.25–0.5) (Figure S1, Supporting Information). Briefly, 1.5 µL chitosan polymer (0.5 mg mL^−1^) and 10 µL chitosan coated iron oxide nanoparticles (CSIONPs) (0.5 mg mL^−1^) were added to a centrifuge tube and the volume of the solution was completed to 500 µL using TAP medium. The mixture was vortexed thoroughly. *C. reinhardtii* at OD_680_ 0.25 was centrifuged for 1.5 min at 300 × *g*. Supernatant was removed and the chitosan/iron oxide NPs mixture was added onto the microalgae pellet gently. *C. reinhardtii* cell suspension was incubated with this mixture for 5 min in an orbital shaker at room temperature. After incubation, microalgae were centrifuged for 1.5 min at 300 × *g* and fresh TAP media was added to the pellet to disperse coated *C. reinhardtii*. After each step of the coating process, algal cells were imaged with an inverted microscope (Nikon Eclipse Ti‐E) for the confirmation of the coatings. Biohybrid algal microswimmers with chitosan and CSIONPs coating were further imaged for 24 h for analysis of stability of the coatings with an inverted microscope (Nikon Eclipse Ti‐E). Images were acquired from the same samples right after coating and 2, 4, 6, and 24 h after coating.

##### Coating Yield of Biohybrid Algal Microswimmers

Coating yield of the biohybrid population was determined by imaging biohybrids coated with red‐fluorescent CSIONPs. Images captured with a Nikon Eclipse Ti‐E inverted microscope using TRITC channel (Ex: 547 nm, Em: 572 nm) were processed by determining the red (coated) algal cells and counting the amount using ImageJ cell counting software. The amount of red (coated) algal cells were divided by the whole amount of algal cells in an image to obtain a coating efficiency. A minimum of three different images were used for the calculation of the coating efficiency (*n* = 3).

##### Construction of the Microfluidic Channels

Swimming speed analysis and light response studies were carried out in microfluidic channels with a height of 350 µm and a width of 2 mm. For the preparation of microfluidic channels, double sided adhesive films and poly (methyl methacrylate) channel pieces were prepared using laser cutter and all components were attached together on a cover glass. Microfluidic channels were treated with 3% Pluronic F‐108 solution for at least 30 min to prevent the nonspecific adhesion of microalgae on the surfaces of the microchannel. Channels were washed with fresh TAP media several times prior injection of microalgae to the channels.

##### Swimming Speed Analysis

For the 2D swimming speed analysis and swimming trajectories of bare and biohybrid microalgae, 2D swimming videos were recorded in microfluidic channels. An in‐house MATLAB code was used to analyze swimming speeds and trajectories of microalgae.

##### Light‐Driven Steering of Bare and Biohybrid Microalgae

For the light‐driven steering of bare and biohybrid microalgae, swimming experiments were performed in microfluidic channel with an axial length of 2 cm. Microfluidic channels filled with microalgae cells were illuminated with white light from right side of the channel for 10 min using the white light source of a microscope (Zeiss Axio Observer A1, Carl Zeiss). Images of left and right side of the channel were captured at 10 min. Next, left side of the channel was illuminated in a similar manner for 10 min and images of both ends of the channel were captured. Quantification of microalgae at the both ends of microfluidic channel was done by using ImageJ cell counting software. Three different images were used for the quantification of the microalgae after light‐driven steering (*n* = 3).

##### SEM Imaging and Energy‐Dispersive X‐Ray Spectroscopy (EDX)

Both bare and biohybrid microalgae were placed on silicon wafers for 1 h to promote the attachment to the surface. Cells were fixed using 2.5% (v/v) glutaraldehyde in 0.2 m sodium cacodylate buffer (pH 7.4) for 40 min at 4 °C. Next, they were rinsed with sodium cacodylate buffer (pH 7.4) for 3 times and dehydrated with ethanol for 10 min using increasing concentrations of ethanol solutions (30%, 50%, 75%, 90%, and 100%). Microalgae were then chemically dehydrated with increasing concentrations of hexamethyldisilazane solutions in ethanol. Samples were dried at room temperature overnight. After air‐drying, samples were coated with 10 nm gold using Leica EM ACE600 sputter coater (Leica Microsystems, Wetzlar, Germany) and electron micrographs were captured with Zeiss Ultra 550 Gemini scanning electron microscope (Carl Zeiss Inc., Oberkochen, Germany) with an accelerating voltage of 5 keV and an in‐lens detector. SEM images were pseudocolored using Adobe Photoshop Software (version 21.0). EDX (Bruker, Billerica, MA) analysis was performed for the detection of magnetic nanoparticles on the surface of microalgae using an accelerating voltage of 15 keV.

##### Conjugation of Photocleavable Linker and DOX to CSIONPs

Photocleavable *o*‐nitrobenzyl linker (1‐(5‐methoxy‐2‐nitro‐4‐prop‐2‐ynyloxyphenyl) ethyl *N*‐succinimidyl carbonate) was conjugated to the amino groups on chitosan coated surface of iron oxide NPs (nonfluorescent) via NHS‐amine reaction. Briefly, 5 × 10^−3^ m linker solution was prepared in dimethyl sulfoxide (DMSO) and mixed with 1 mg mL^−1^ CS‐iron oxide NP solution for 2 h at room temperature by vortexing. Next, the particles treated with photocleavable linker were reacted with a solution containing 0.1 × 10^−3^ m DOX, 0.1 × 10^−3^ m CuSo_4_, 5 × 10^−3^ m sodium ascorbate, and 0.5 × 10^−3^ m tris(3‐hydroxypropyltriazolylmethyl)amine for 2 h at room temperature by vortexing. DOX loaded CS‐iron oxide NPs (CSIOPNs‐L‐DOX, L: linker) were washed several times with ddH2O and DMSO to remove unreacted molecules.

##### Characterization of CSIONPs after Drug Conjugation

Hydrodynamic diameters of CSIONPs and CSIONPs‐L‐DOX were measured with DLS technique (Möbius, Wyatt Technologies). CSIONPs‐L‐DOX were stored at 4 °C until further use. For the determination of the unbound DOX amount after conjugation reaction, particles with (CSIONPs‐L‐DOX) and without linker (CSIONPs‐DOX) were washed three times after DOX reaction. After each washing step, particles were spun down for 1 h at 30 000 × *g* and supernatants were collected for fluorescence measurements. Additionally, fluorescence intensities of CSIONPs‐DOX and CSIONPs‐L‐DOX were measured with a plate reader at Ex: 485 nm, Em: 528 nm (BioTek's Synergy H1, Winooski, VT, USA).

##### Light‐Triggered DOX Release from CSIONPs

DOX release from CSIONPs were achieved by illuminating nanoparticle solution (1 mg mL^−1^) with 365 nm UV light (45 mW cm^−2^) for 5 s. After light‐triggered release, nanoparticles were vortexed for 2 h, centrifuged at 30 000 × *g* for 1 h and DOX concentration in supernatant were measured by reading the fluorescence of the samples with a plate reader at Excitation: 485 nm, Emission: 528 nm (BioTek's Synergy H1, Winooski, VT, USA).

##### Cell Culture

SK‐BR‐3 cells were purchased from ATCC. Cells were cultured in McCoy's 5a Medium Modified, supplemented with 10% FBS at 37°C and 5% CO_2_ incubator. Medium was refreshed every 4 days and cells were subcultured once a week.

##### DOX Release from Biohybrid Microalgae and DOX Internalization

For incubation of microalgae with SK‐BR‐3 cells, cells were seeded in microwells with flat glass bottoms (ibidi, Martinsried, Germany). 25 × 10^3^ cells per well were plated and after reaching ≈80% cell confluency (after 72 h), bare microalgae were added to the culture in a 1:1 mixture of McCoy's 5a Medium:TAP Medium. For the evaluation of the effect of bare microalgae concentration on cell viability, microalgae dispersions at different OD_680_ values (0.6, 0.8 and 1.0) were incubated with SK‐BR‐3 cells for 2 or 24 h at 37 °C and 5% CO_2_ incubator. At the end of the incubation, a colorimetric cell counting assay (WST‐8) was performed to evaluate cell viability. Briefly, 10% WST‐8 solution was prepared with cell culture media and cells were incubated with this solution for 1 h at 37 °C and 5% CO_2_ incubator. Next, absorbance at 460 nm was measured. Since the highest cell viability was observed in the case of OD_680_: 0.6, further experiments with bare and biohybrid microalgae were performed with this OD value.

For DOX release from biohybrid microalgae experiments, 300 µL biohybrid microalgae dispersion was placed on a monolayer of SK‐BR‐3 cells and illuminated with 45mW cm^−2^ UV light for 5 s. Biohybrid microalgae and SK‐BR‐3 cells were incubated together for 2 h at 37 °C and 5% CO_2_ with gentle shaking every 30 min to allow dispersion of the released drug thoroughly. Additionally, cells were imaged with an inverted epifluorescence microscope (Nikon Eclipse Ti‐E) and DOX internalization was investigated.

##### Statistical Analysis

Swimming speed and trajectory results were calculated by averaging at least 5 independent motility analyses. Statistical analyses were performed using unpaired *t*‐test and all quantitative values in graphs were presented as means ± standard error of the mean (SEM). *P* < 0.05 was considered statistically significant.

## Conflict of Interest

The authors declare no conflict of interest.

## Supporting information

Supporting Information

Supplemental Video 1

Supplemental Video 2

Supplemental Video 3

Supplemental Video 4

Supplemental Video 5

Supplemental Video 6
