# Supplementary material for: High‐Yield Production of Biohybrid Microalgae for On‐Demand Cargo Delivery
Source: Adv Sci (Weinh). 2020 Jul 2;7(16):2001256. doi: 10.1002/advs.202001256 (PMC7435244; doi:10.1002/advs.202001256)
Supplement: Supplementary file 1 — Supporting Information [file ADVS-7-2001256-s001.pdf]

Supporting Information

for

High-Yield Production of Biohybrid Microalgae for On-Demand Cargo Delivery

Mukrime Birgul Akolpoglu[1,*], Nihal Olcay Dogan[1,2,*], Ugur Bozuyuk[1,*], Hakan Ceylan[1,†], Seda Kizilel[2,†], Metin Sitti[1,3,4,†]

[1] Physical Intelligence Department, Max Planck Institute for Intelligent Systems, 70569 Stuttgart, Germany

[2] Chemical and Biological Engineering Department, Koç University, 34450 Istanbul, Turkey

[3] School of Medicine and School of Engineering, Koç University, 34450 Istanbul, Turkey

[4] Institute for Biomedical Engineering, ETH Zurich, 8092 Zurich, Switzerland

[*] Equally contributing first authors

[†] Correspondence to: sitti@is.mpg.de, ceylan@is.mpg.de, skizilel@ku.edu.tr

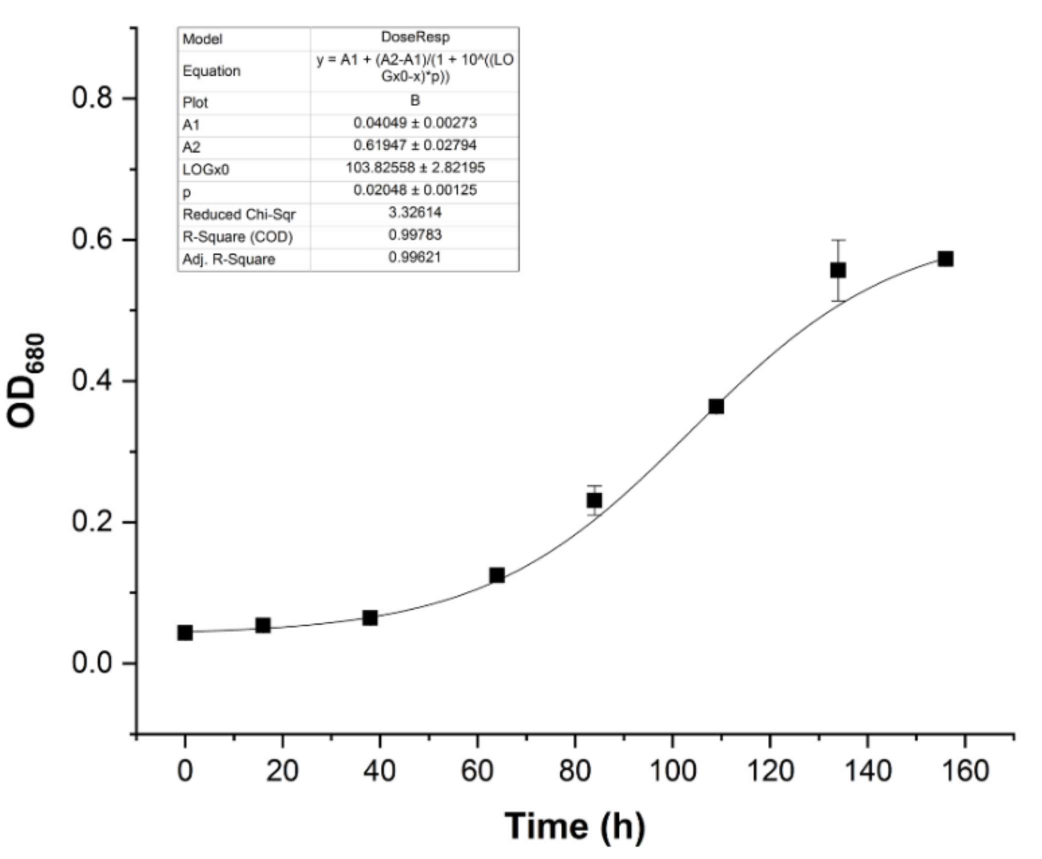

Figure S1. Growth curve of *C. reinhardtii*.

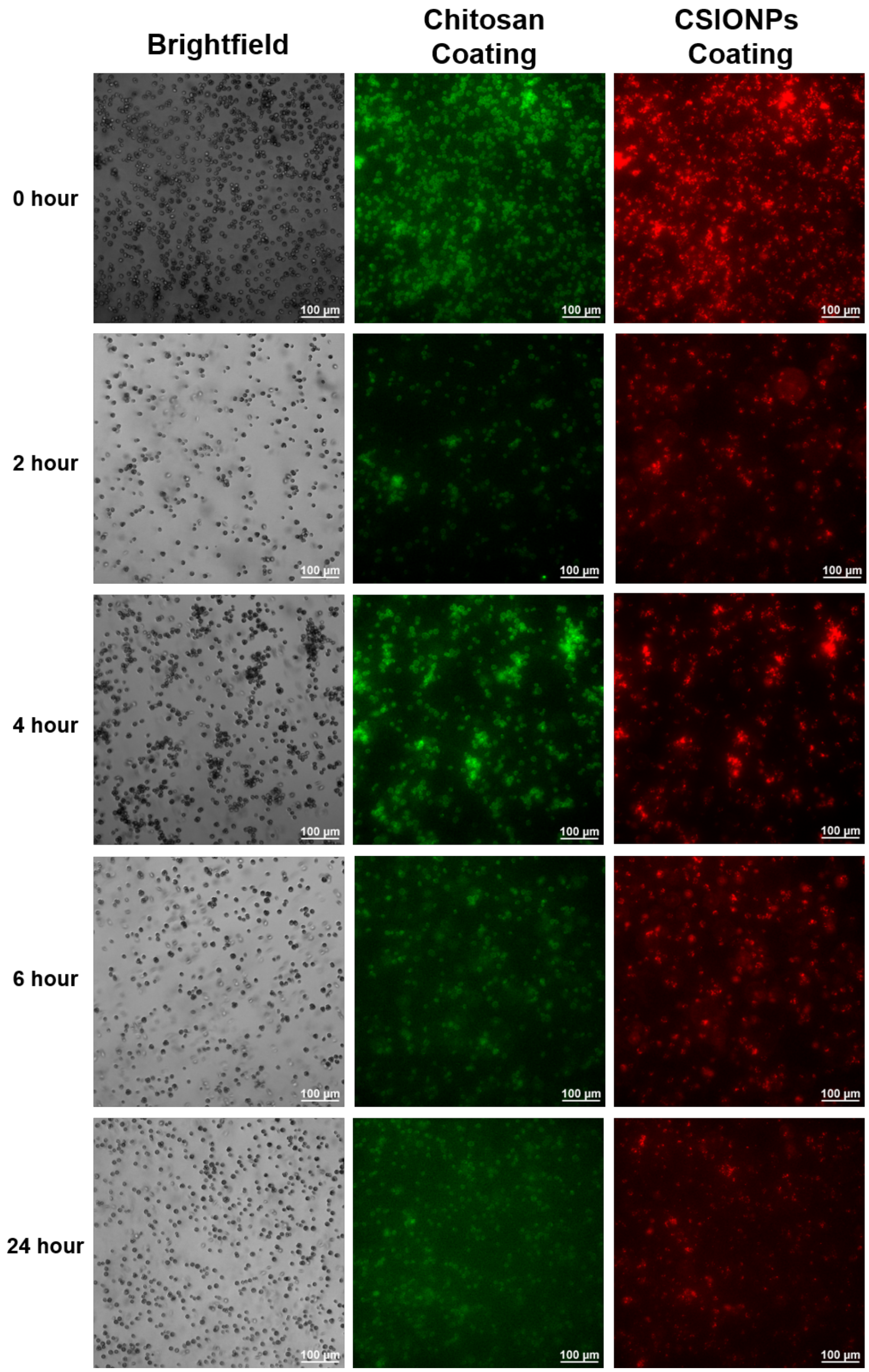

Figure S2. Microscopy images showing the time stability of chitosan polymer and CSIONPs coating on microalgae. Images were captured at time points t=0 h, 2 h, 4 h, 6 h, and 24 h. Scale bars = 100 µm.

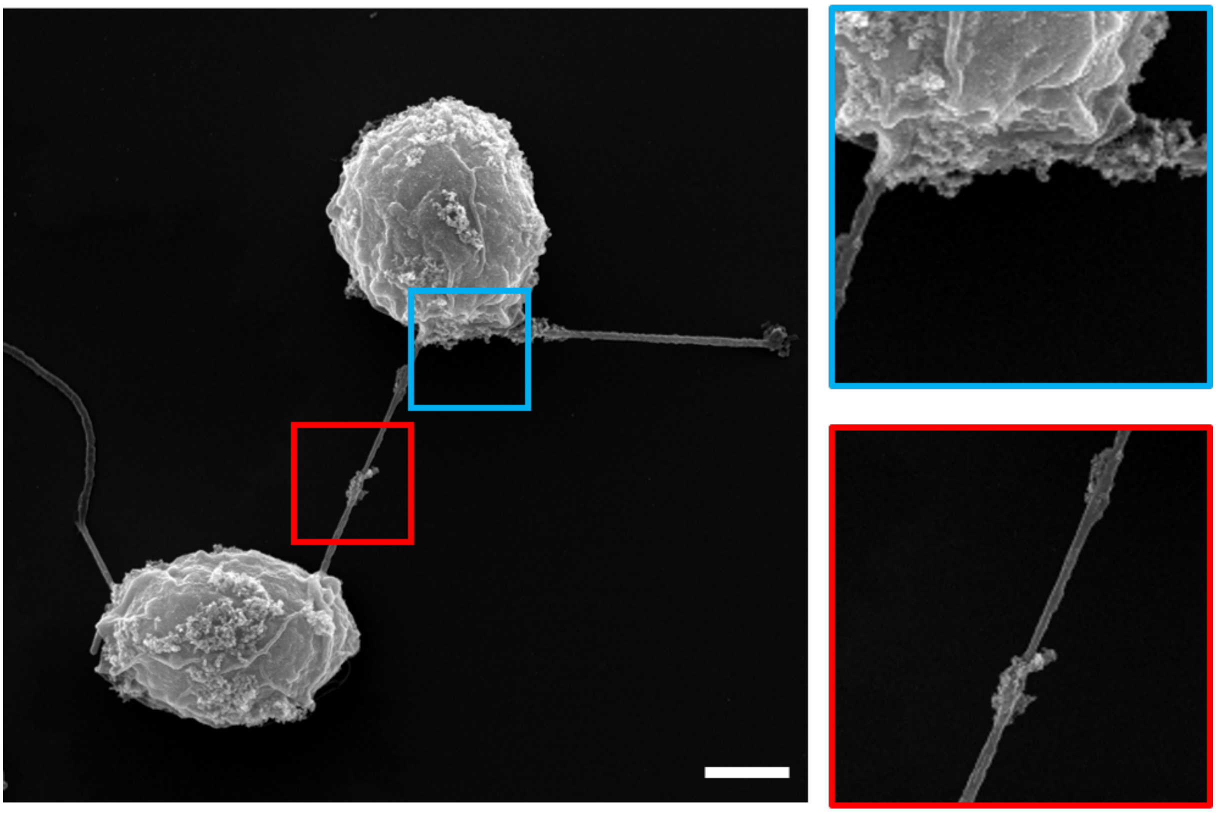

Figure S3. SEM image of example biohybrid microalgae. Blue and red framed close-ups represent the attachment of CSIONPs on or close to the flagella. Scale bar = 2 µm.

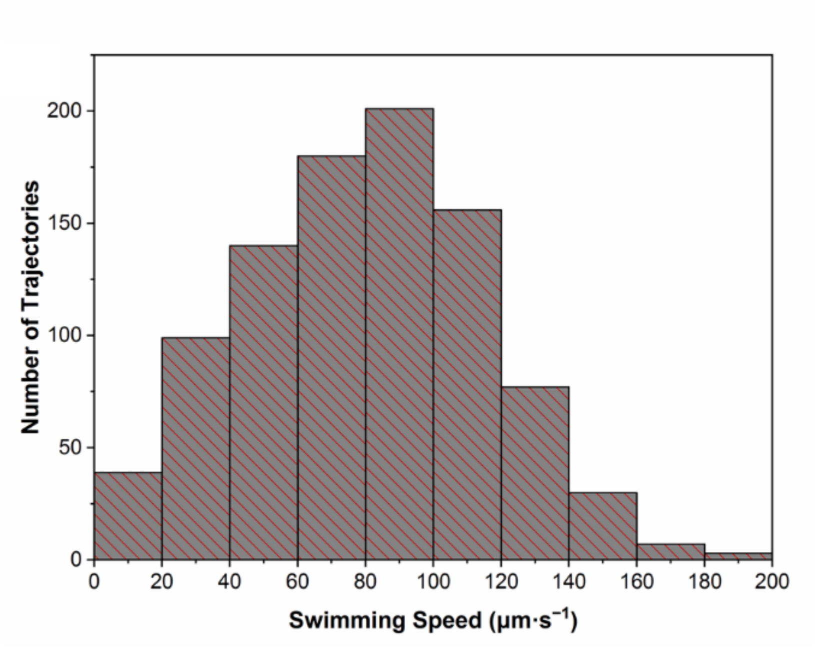

Figure S4. Swimming speed measurements of bare microalgae centrifuged twice at 300 x g for 1.5 min. Average swimming speed is 80 μm/sec.

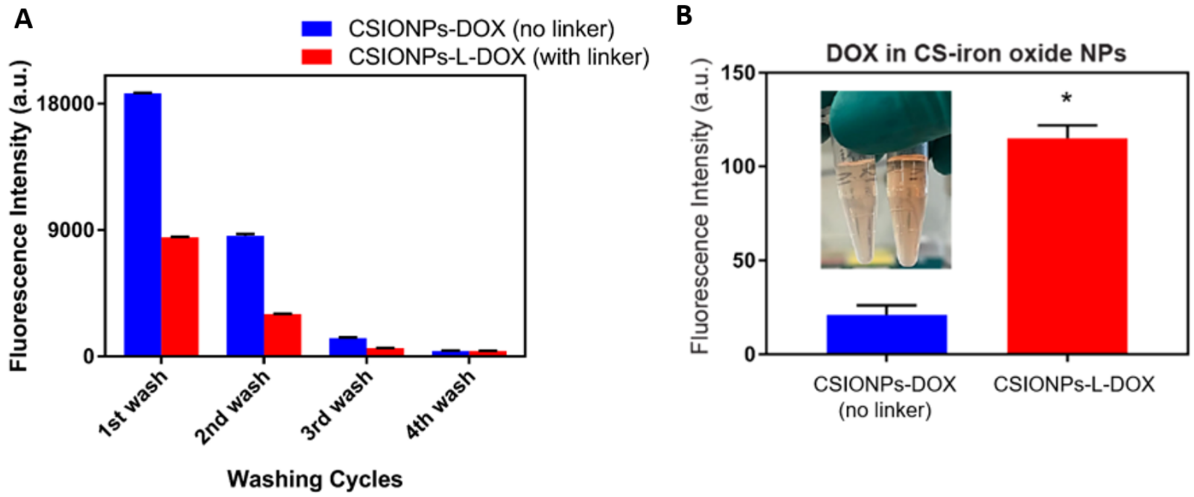

Figure S5. Characterization of DOX conjugation on CSIONPs through *o*-nitrobenzyl photocleavable linker. A) Fluorescence of supernatants were measured after each washing step. Fluorescence intensity of CSIONPs-DOX (no linker) showed higher fluorescence, indicating the absense of a chemical conjugation between DOX-azide and CSIONPs. B) Fluorescence intensity of CSIONPs-L-DOX modified nanoparticles.

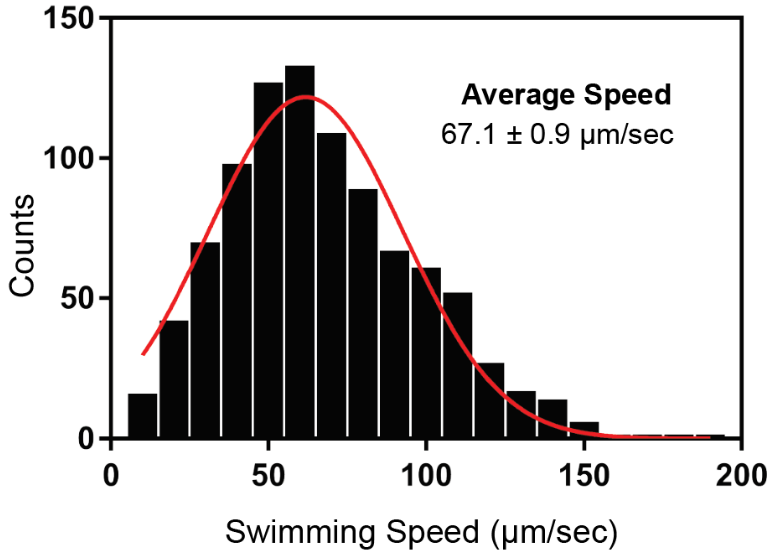

Figure S6. Swimming speed experimental data of CSIONPs-L-DOX-modified biohybrid microalgae.

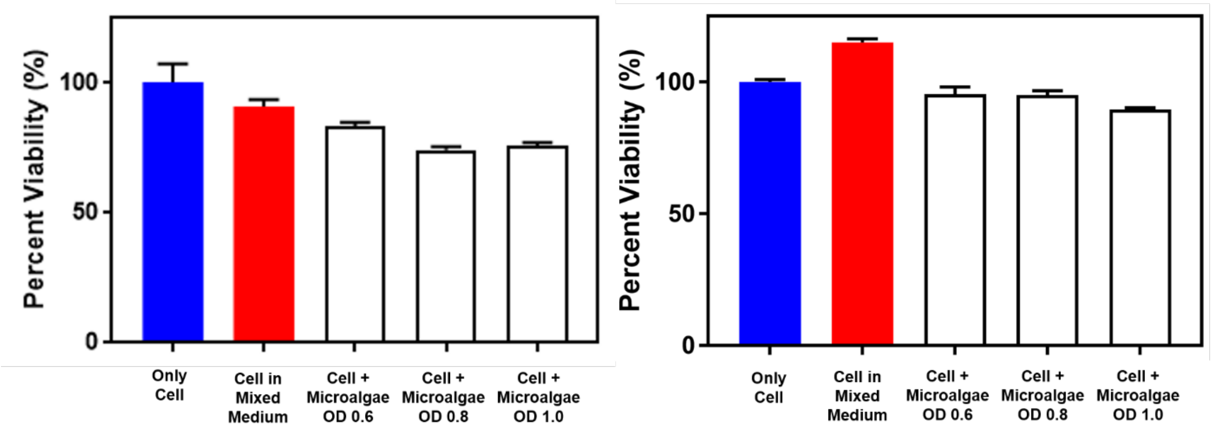

Figure S7. Percent viabilities of SKBR-3 cells after incubation for 2 h with bare microalgae at different OD values. A cell counting assay (WST-8) was performed 24 h (left) and 48 h (right).

Supplementary Movies

Movie S1. Swimming of bare microalgae in TAP medium.

Movie S2. Swimming of biohybrid microalgae in TAP medium.

Movie S3. Swimming of biohybrid microalgae showing chitosan coating in TAP medium.

Movie S4. Swimming of biohybrid microalgae showing CSIONPs coating in TAP medium.

Movie S5. Swimming of bare microalgae centrifuged twice.

Movie S6. Direct contact of biohybrid microalgae with SK-BR-3 tumor cells after DOX release.
